# Salt Crystallization in Limestone: Materials Decay and Chemomechanical Approach

**DOI:** 10.3390/ma17163986

**Published:** 2024-08-11

**Authors:** Marta Cappai, Marta Casti, Giorgio Pia

**Affiliations:** 1 Dipartimento di Ingegneria Meccanica, Chimica e dei Materiali, Università degli Studi di Cagliari, Via Marengo 2, 09123 Cagliari, Sardinia, Italy; marta.cappai@unica.it; 2 Materialia Association, 09037 San Gavino Monreale, Sardinia, Italy; mcasti87@gmail.com

**Keywords:** limestone, sodium sulfate, decay

## Abstract

Salt crystallization is a particularly relevant issue in the conservation of limestones used in Cultural Heritage sites. In this study, various facies of limestones were characterized through porosimetric and mechanical tests. The samples were subjected to experiments to determine their resistance to salt crystallization by verifying the number of cycles at which 50% of them began to lose weight. This number of experimental cycles was compared with the result calculated by the analytical procedure of a chemomechanical model found in the literature. The comparison showed a significant capability of the model to predict the experimental data.

## 1. Introduction

Porous materials are an important class in the construction and building field. Indeed, regardless of their origin, natural or artificial, most of the materials used in construction are porous. The presence of voids in the microstructure, on the one hand, can be a positive aspect—for instance, reducing the density of materials and improving thermal insulation—while on the other hand, it can be a detrimental factor for mechanical properties and durability [1,2,3,4,5]. This latter characteristic becomes particularly significant, especially when discussing materials used for the preservation of Cultural Heritage.

Water circulation into the porous structure and capillary absorption can contribute to different weathering processes such as the dissolution of soluble phases, biological colonization, freeze–thaw decay, and damage from salt crystallization cycles [1,6,7,8,9,10,11,12,13]. This last is one of the main forms of degradation affecting in situ materials such as ceramic bricks, cements, or building stones and is the deterioration caused by the crystallization cycles of salts and the related expansive and contraction phenomena [14,15,16,17,18,19,20,21,22,23,24,25,26,27,28].

Specifically, the percolation of aqueous solutions from external sources (such as rainwater or soil water), aerosol deposition, or the reaction of stone minerals with atmospheric substances introduces soluble salts into the porous microstructure [19,26,29,30]. The accumulation of these salts increases due to water evaporation phenomena. When the solution becomes supersaturated with respect to a specific salt phase, it precipitates, leading to phenomena known as efflorescence and subflorescence. Efflorescence occurs with a slow rate of evaporation, allowing the aqueous solution contained in the pores to reach the surface before evaporating, resulting in surface salt crystallization. In contrast, subflorescence occurs when evaporation proceeds at a considerable rate, causing the aqueous solution to evaporate within the pores, leading to internal salt accumulation and crystallization within the material matrix [19,26,27,29,30]. Relevant examples of salt crystallization damage are represented by Luxor Egyptian construction, the ruins of Petra, Pompeii monuments, etc. [7,21,31,32,33].

The extent of damage is influenced by the type of salt involved in the crystallization and solubilization processes [26,28]. Some salts are particularly hygroscopic, and their phase changes are closely linked to the environmental conditions they are exposed to. By considering the ions in the solutions, a large number of salts (more than 85) can be found in stones. Some of them are relatively common, such as sulfate, chloride, sodium, potassium, magnesium, nitrate, and calcium, while others are less common, such as carbonate or bicarbonate, phosphate, fluoride, acetate, oxalate, ammonium, nitrite, and formate [25].

One of the most damaging salts is sodium sulfate, which crystallizes in various forms. However, the primary forms found in building materials are thenardite (anhydrous, Na_2_SO_4_) and mirabilite (hydrate, Na_2_SO_4_·10H_2_O). This hydration process consists in a large volume variation (expansion) equal to 314% [30].

Rodriguez-Navarro et al. found that mirabilite crystallizes at relative humidity (RH) levels above 50%, while thenardite crystallizes at RH levels below 50% [17,22]. 

Lopez-Arce et al. [17] performed a series of experiments on sodium sulfate crystallization observed by ESEM in a climatic chamber. They determined that the critical RH value is around 75%, which represents the inflection point for volume variations (expansion/contraction and hydration/dehydration reactions). At this point, expansion occurs as humidity increases above 75% RH, and contraction occurs as humidity decreases below 75% RH, and both are accompanied by corresponding volume changes. These authors, considering the crystallization rate and the speed of humidity variation, emphasize that thenardite plays a significant role in masonry decay [17], while other authors have identified mirabilite as the primary cause of degradation [29].

Chatterji and Jensen [34] investigated the solubility of thenardite and mirabilite under varying relative humidity conditions, observing that the solubility of the anhydrous phase is significantly higher than that of the hydrate below 32 °C. Under these conditions, any circulation of aqueous solutions within the structure of a porous material or an increase in relative humidity will lead to the dissolution of thenardite and the precipitation of mirabilite [34]. The cyclical repetition of these events (imbibition/drying) results in an accumulation of precipitate and a significant increase in tensile stress, exceeding the allowable stress limits of the material, leading to its decay and a consequent loss of weight.

These degradation processes are particularly harmful to limestone. This fact becomes even more significant when considering the widespread use of this stone and the considerable historical and artistic heritage created over the centuries [35,36,37,38,39,40]. Furthermore, studying these materials in situ at Cultural Heritage sites is even more complex due to the impossibility of performing large-scale sampling, which is necessary for material characterization. This has led to intense laboratory activity on facies similar to those found in Cultural Heritage objects and highlights the ambitious goal of developing analytical procedures capable of predicting the harmfulness of certain conditions over others. In this way, it will be easier to implement appropriate conservation strategies.

In this study, the resistance of Miocene limestone was evaluated through experimental tests involving the imbibition of a sodium sulfate solution and the drying of the samples. Specifically, as the cycles of the test (imbibition and drying) progressed, the weight loss of the samples (relative to the initial weight) was measured. This weight loss is attributed to the mechanical stresses induced by the cyclic phenomenon of salt crystallization and subsequent dissolution as hygrometric conditions vary. It serves as an indicator of the degradation that porous materials of this nature can experience, potentially leading to significant and compromising levels of destruction, which, in macroscopic terms, put at risk the preservation of Cultural Heritage.

Additionally, a chemomechanical model was applied to predict the service life of the samples in terms of exposure cycles to the sodium sulfate crystallization phenomenon. This model has already proven to be particularly reliable in the original work proposed by Flatt et al. [29]. Specifically, it was possible to calculate the number of salt crystallization cycles after which local stresses generate macroscopic tensions that exceed the material’s tolerable limits (experimentally measured through tensile mechanical tests) [29]. The model assumes that surpassing these limits corresponds to structural damage, resulting in weight loss due to the destruction of the sample [29].

In this work, by comparing the number of cycles at which weight loss is experimentally observed with the number of cycles at which the material’s stress limits are calculated to be exceeded, a substantial agreement is found.

Overall, the aim of this work is twofold. On one hand, the purpose is to verify the resistance of Miocene stone, which is widely used in the Mediterranean basin for the construction of buildings that are now considered Cultural Heritage sites, to sodium sulfate crystallization cycles. On the other hand, the objective is to validate the chemomechanical model to test the reliability of an analytical tool that could be useful in the conservation process.

## 2. Materials and Methods

In this study, several samples of Miocene limestone (specifically from the middle–upper Burdigalian–Langhian) were analyzed, originating from central-western Sardinia (Italy) Santa Caterina di Pittinuri (40°06′27.8″ N 8°30′16.9″ E). This rock, characteristic of the limestone cliffs in the area, features numerous bioclastic fragments and a homogeneous and porous texture. The importance of this limestone lies in its historical use in various archaeological sites across Sardinia [41].

The Bruker D8 Advance diffractometer (Leipzig, Germany), characterized by a multimode LYNXEYE XE-T detector, has been used for obtaining X-ray analysis and relative mineralogical phases which constituted the materials. Our tests employed Cu Kα radiation, scanning over a range of scattering angles (2θ) from 5° to 80°, with a step size of 0.05° and an acquisition time of 15 s per angle. The obtained patterns were analyzed using Materials Analysis Using Diffraction (MAUD) software [42,43].

Micromeritics^®^ Autopore IV 9500 Porosimeter has been used to perform Mercury Intrusion Porosimetry (MIP) tests (conditions: 2200 bars; equilibration time: 10 s). Each measurement was repeated three times per sample. The sample dimensions were approximately 1.5 cm^2^.

Mechanical tests were performed to measure tensile stress by using an MTS Landmark 370 (Eden Prairie, MN, USA) universal test machine. 

Six series, each consisting of 8 samples, were individuated as a function of porosity. Cubic samples with a side length of 40 mm were used. For the determination of resistance to salt crystallization, the samples were oven-dried to a constant mass and then immersed in a sodium sulfate solution with a concentration of 0.0645 ± 0.0001 g·mL^−1^ for 2 h at 25 °C. After two hours, the samples were dried in the oven at 105 °C for twenty hours and then cooled to room temperature. This procedure was repeated for 15 cycles. At the end of each cycle, the samples were weighed to monitor any weight gain or loss.

## 3. Results and Discussion

**Figure 1:**
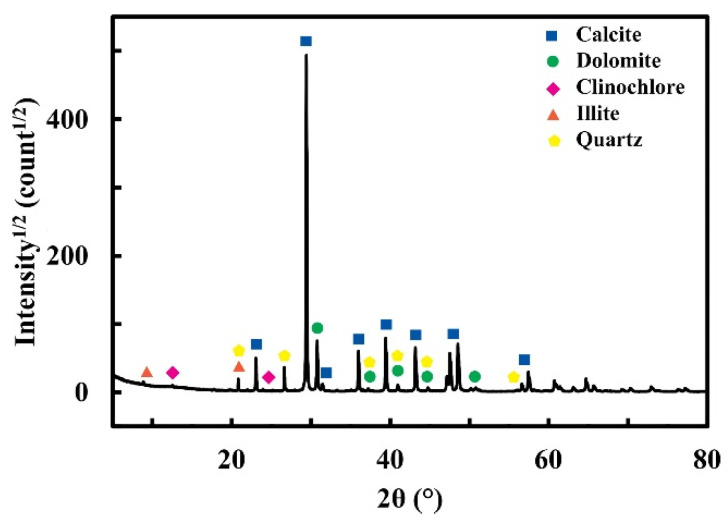
X-ray diffraction pattern of Miocene limestone.

Six different facies of Miocene limestones have been characterized to study their decay caused by sodium sulfate crystallization. XRD analysis, performed using the Rietveld method, shows that the mineralogical compositions are very similar for each system and are composed of the following: calcite: 69.93 ± 0.00%; dolomite: 28.11 ± 0.69%; clinochlore: 1.28 ± 0.20%; illite: 0.29 ± 0.22%; quartz: 0.38 ± 0.02%. A typical XRD pattern is reported in Figure 1.

**Figure 2:**
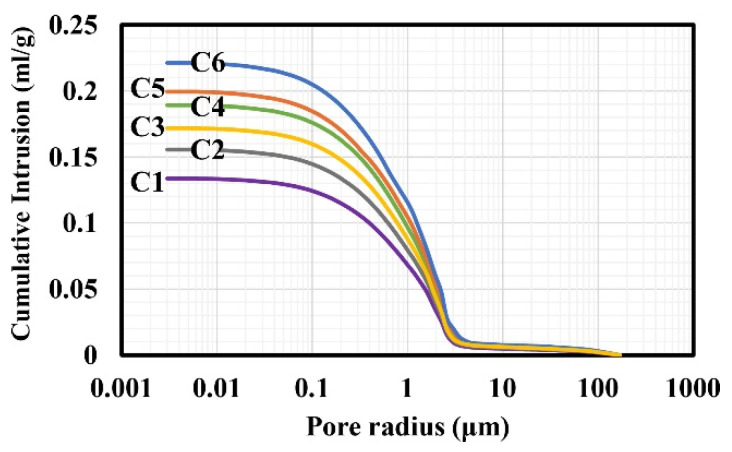
Pore cumulative curves for different series of Miocene limestones studied (**—** C1; **—** C2; **—** C3; **—** C4; **—** C5; **—** C6). In particular, considering three different pore radius ranges in relation to the hazard level of salt crystallization (with the highest risk identified in the range between 1 and 0.05 μm [1,3,44]), it can be observed that approximately 50% of the porosity is distributed within the high-risk vulnerability range. Indeed, such pore size distributions lead to high crystallization pressures that can cause significant damage to the material’s microstructure [22]. Consequently, the average pore radius (APR) of the distribution falls within the critical range, with values between 0.322 μm and 0.185 μm.

In Figure 2, cumulative curves relative to different systems, obtained using the MIP technique, are reported. It is notable that the pore size range is between 100 μm and 0.01 μm. The inflection point, which corresponds to the maximum pore size distribution, is around 2 μm. As reported in Table 1, the average pore volume fraction (ε) is specific for each facies and ranges between 43.37% ± 2.00 and 26.00% ± 2.00 (density δ between 1.58 and 1.96 g/cm^3^). This variability is due to the significant heterogeneity of this type of sedimentary rock and the relative diagenetic conditions and processes.

The specific surface area values (SSAs), which range from 1.91 to 1.14 m^2^/g, are directly proportional to the porosity and indicate an increasing risk of salt crystallization within the porous microstructure. This is due to the presence of a high number of nucleation sites that accelerate and intensify the process [1,3,17,44].

In Table 1, the respective values for tensile strength (σT) for different limestone facies are also reported, ranging from 4.0 ± 0.2 MPa to 2.6 ± 0.6 MPa. As expected, these variations are inversely proportional to porosity data.

The observation of porosimetric and mechanical data highlights an increasing risk of degradation from system C1 to C6, which must be confirmed through salt crystallization resistance tests.

**Figure 3:**
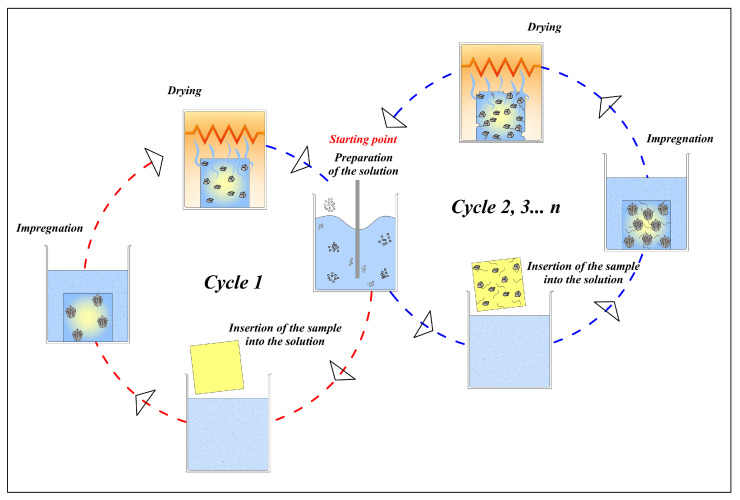
Scheme of experimental procedure for the determination of resistance to salt crystallization. The starting point of each cycle coincides with the preparation of the solution.

The type of experiment is summarized in Figure 3. During a typical test cycle, the dry sample is weighed and subsequently imbibed with an aqueous sodium sulfate solution. Once the absorption is complete (which takes 2 h at 25 °C), the sample is dried in an oven (20 h at 105 °C) and then cooled to room temperature. A subsequent weight measurement initiates the new cycle, recording any mass variations. 

**Figure 4:**
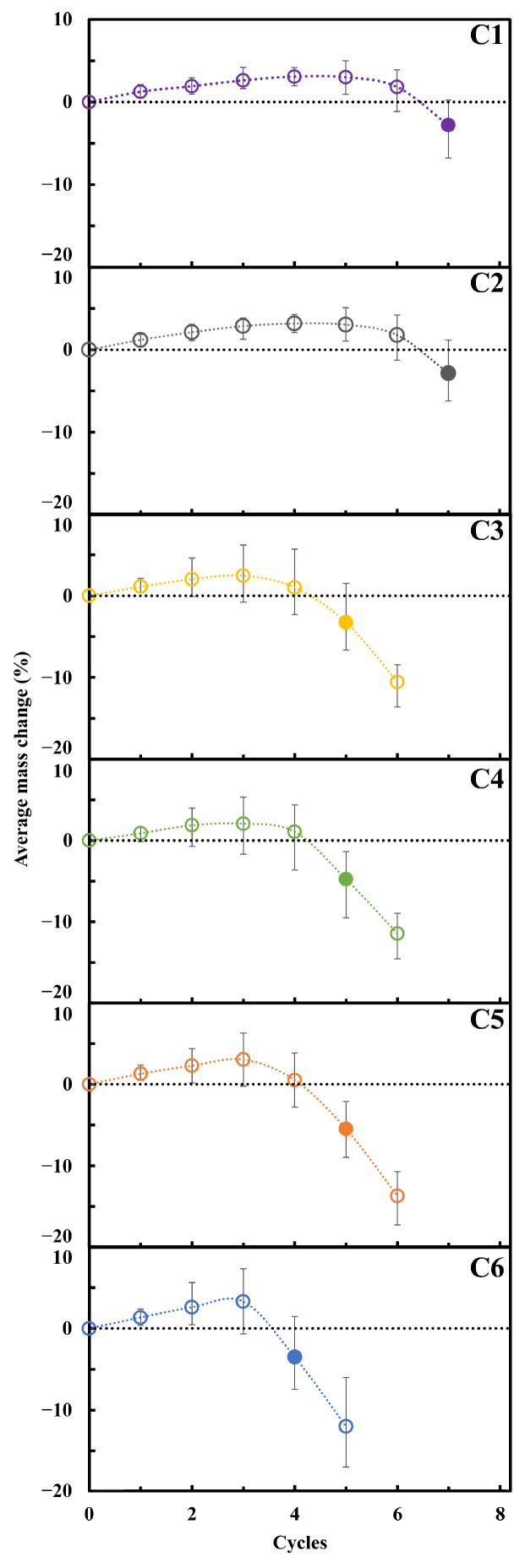
Mass variation of studied limestones during salt crystallization test. Filled points correspond to the cycles in which 50% of samples have lost mass.

Figure 4 reports mass variation during the experiment. Initially, the samples increase in weight due to the accumulation of sodium sulfate (thenardite) in the pore structure.

The conditions occurring in the subsequent cycles involve a process of thenardite dissolution, leading to a state of sodium sulfate supersaturation and subsequent precipitation in the form of mirabilite. The crystallization of mirabilite generates high pressures within the porous matrix, resulting in cracking and ultimately the fracturing of the material, the consequent loss of parts, and therefore a decrease in the sample’s mass.

It can be observed that the samples lose weight (relative to their initial masses) after a different number of cycles. Their resistance to crystallization is correlated with their porosity and tensile strength. In fact, the samples that exhibit weight loss after a higher number of cycles belong to systems with lower porosity and higher tensile strength. Conversely, the weaker samples have significant porosity and lower tensile strength.

In the adopted experimental procedure formalized by Flatt et al. [29], the starting point of considerable degradation corresponds to the cycles in which 50% of the samples have lost mass, represented in the graphs as filled points.

The ability to study and predict this phenomenon and its effects is of fundamental importance in the field of Cultural Heritage. 

The severity of the decay is related to crystallization pressure, which can be expressed as follows:

(1) $$∆P_{C}=\frac{RT}{{\bar{\nu }}_{M}}\left[K_{sp,T}-K_{sp,M}+10ln\;\left(\frac{{RH}_{sat,T}}{100}\right)\;\right]$$

where *R* (8.314 J∙mol^−1^∙K^−1^) is the gas constant, *T* (298.15 K) is the temperature, and ${\bar{\nu }}_{M}$ is the molar volume of mirabilite (217.7 cm^3^ mol^−1^). *K_sp,T_* and *K_sp,M_* denote the thermodynamic solubility products of the two considered phases of sodium sulfate: thenardite and mirabilite, respectively [45]. *RH_sat,T_* represents the relative humidity at which thenardite begins to absorb moisture from the air and starts to transition into a liquid solution (deliquescence). It is determined by accounting for water activity in a nonideal solution, utilizing the Pitzer parameter suited for relevant concentrations [45].

Starting from this consideration, Flatt et al. [29] proposed a model based on a poro-mechanical approach in which mirabilite is assumed to be homogeneously distributed into the microstructure of the sample. By analyzing a characteristic volume, the applied average stress, which corresponds to the macroscopic tensile stress, can be analytically expressed as follows:

(2) $$\sigma^{*}=\sigma_{r}bS_{c}$$

where *b* (0.74 in this work) represents the Biot coefficient; *σ_r_* is the radial compressive strength which can be equated to Δ*P_c_* (Equation (1)); and *S_c_*, derived from the analytical formula, is expressed as follows:

(3) $$S_{c,i}=\frac{{\bar{\nu }}_{T}}{{\bar{\nu }}_{M}}\left[1-{\left(1-{\bar{\nu }}_{T}\frac{c_{{Na}_{2}{SO}_{4}}}{M_{T}}\right)}^{i}\right]$$

Equation (3) allows the calculation of the volume fraction of the pore space filled with crystals, taking into account that ${\bar{\nu }}_{T}$, *M_T_*, $c_{{Na}_{2}{SO}_{4}}$ are the molar volume of thenardite (53.3 cm^3^·mol^−1^); the molar mass of thenardite (142.04 g·mol^−1^); and the concentration of sodium sulfate in the solution (0.0645 ± 0.0001 g·mL^−1^). Under these conditions, *S_c_* is approximately one, indicating full pore saturation, after 11–12 cycles.

Critical stress for specific materials is calculated by using a strain energy criterion, as follows:

(4) $$\sigma_{C}^{*}=\frac{\sigma_{T}}{\sqrt{3\left(1-2\nu \right)}}$$

It represents the values at which the material is expected to fail, where *ν* (0.28 ± 0.02) is the Poisson’s ratio and *σ_T_* is the experimental tensile strength (Table 1, tensile strength for different facies). 

**Figure 5:**
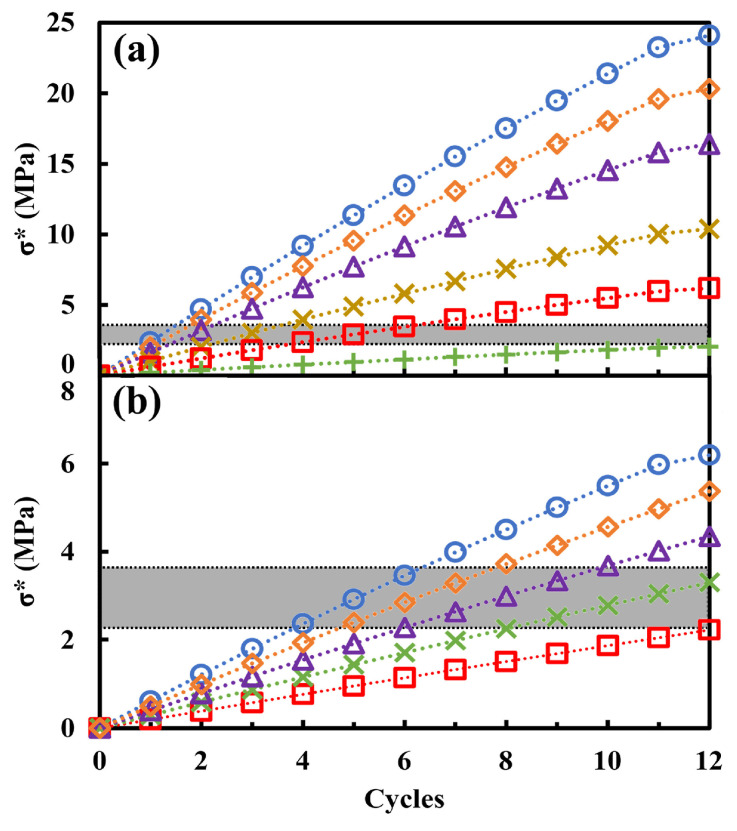
(**a**) σ* at different temperatures (**○** = 2.5 °C; **◊** = 7.5 °C; **Δ** = 12.5 °C; **×** = 20 °C; **□** = 25 °C; **+** = 30 °C) with a concentration of 6.1% sodium sulfate in water; (**b**) σ* at different concentrations (**○** = 6.1%; **◊** = 5%; **Δ** = 4%; **× **= 3%; **□** = 2%) at a temperature of 25 °C. The gray band indicates the critical stress range between 2.17 and 3.65 MPa.

A parametric inspection has been performed by considering different conditions in which systems could be found (Figure 5). In particular, a case was considered where the temperature varies (2.5, 7.5, 12.5, 20, 25, and 30.0 °C) and consequently, there are changes in the RH value and the solubility products of the sodium sulfate mineralogical phases (thenardite and mirabilite) [45], and a case was considered where the concentration of the solutions varies (2, 3, 4, 5, 6.1% *w*/*w*) while keeping the temperature constant (T = 25 °C). These calculations show how both quantities are capable of significantly influencing salt crystallization and thus of establishing the potential danger of the phenomenon.

The effects (degradation) of the macroscopic tensile stresses generated by salt crystallization are consequent to the value of the critical stress of the specific material.

In the case of the limestones considered, the critical stress ($\sigma_{C}^{*}$) varies between 2.17 and 3.65 MPa for the different systems (gray band in Figure 5). In general, it can be seen that in both the case where the temperature reaches 30 °C, as already observed by Price [29], and the case where the concentration is less than 2%, the limestone does not fail. In fact, the stress value due to salt crystallization in the porous structure of the sample does not exceed the material’s limiting strength. This set of data confirms that the phenomenon studied is particularly damaging within a range of temperatures and concentrations that frequently occur in the environmental conditions in which the materials are used.

Thus, the model has the purpose and ambition of determining the number of salt crystallization cycles after which sample damage and subsequent weight loss occur.

**Figure 6:**
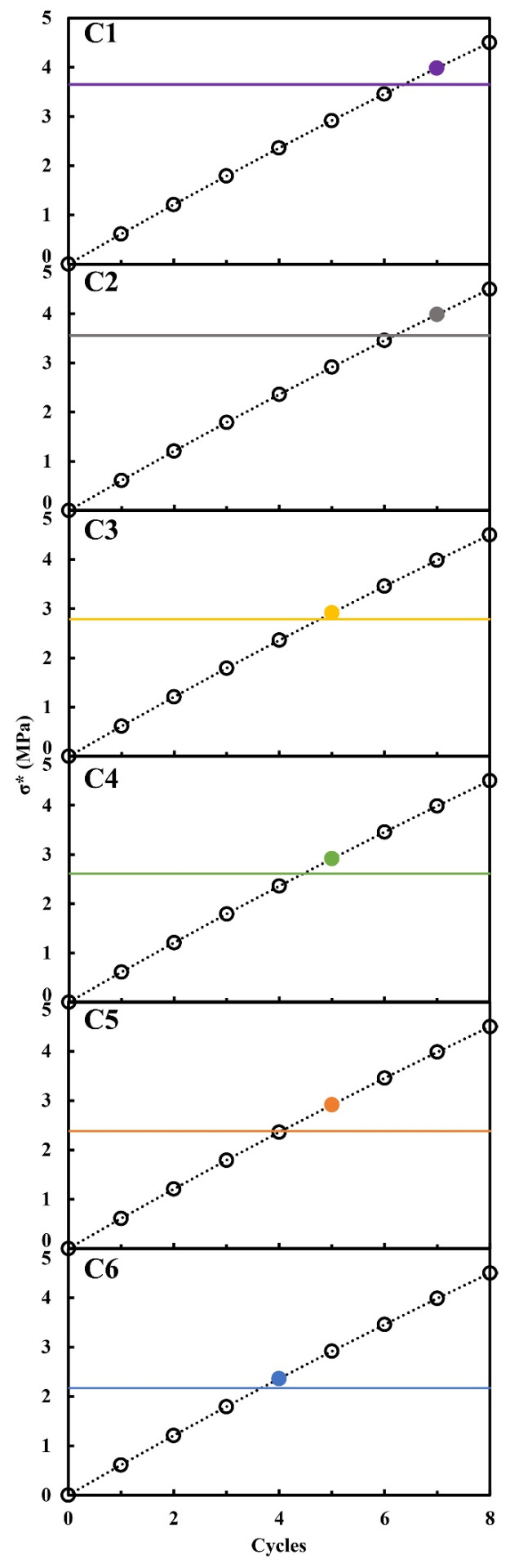
Model calculations for predicting macroscopic tensile strength and comparison with critical macroscopic tensile strength. Filled points represent the value for which 50% of samples have lost mass. Calculations have been performed for T = 25 °C, at a concentration of 0.0645 ± 0.0001 g·mL^−1^.

Figure 6 reports calculations performed using Equation (2) for macroscopic tensile strength and Equation (4) for critical stress for each system. From the assumptions made, it is calculated that the cycle corresponding to the occurrence of sample degradation (weight loss with respect to the initial mass) is derived from the projection on the x-axis of the first tensile stress value greater than the critical stress value.

To verify the reliability of the model, it is necessary, as proposed by Flatt et al. [29], to compare the calculated number of cycles at which material degradation occurs with the number of cycles at which at least 50% of the samples in a series begin to lose weight compared to their initial mass.

The cycle value at which weight loss (and thus degradation) from salt crystallization is experimentally observed in Figure 4 is given in Figure 6 (filled point).

A comparison between the experimental results and calculations shows that the model is capable of predicting the cycle in which samples start to lose mass and, consequently, predicting the decay process. 

It is evident that porosity has a significative influence. Indeed, samples which have a greater pore volume fraction lose mass after fewer cycles compared to those that are less porous. It is necessary to consider that not only does porosity increase in the different samples that lose a greater amount of material, but specifically, the fraction of pores within the range of critical radii for the phenomenon of salt crystallization increases [3]. Conversely, given the inverse proportionality between porosity and mechanical properties, the number of cycles the material can resist before weight loss is inversely proportional to the tensile strength.

This result validates the adopted modeling approach and provides an easily applicable analytical procedure for predicting degradation due to salt crystallization [29]. 

The formalization of new reliable analytical procedures is of fundamental importance in the field of Cultural Heritage degradation, where extensive sampling of materials in situ is not possible due to the need to protect the property. Therefore, acquiring experimental information through minimal sampling (such as that required for XRD and MIP tests) is essential to obtain data on the nature of the material and its microstructure. Using these data as input (both direct and indirect) for modeling can offer the possibility of calculating physical quantities and resistance to degradation that could otherwise only be estimated through statistically robust experimental campaigns.

## 4. Conclusions

The determination of resistance to salt crystallization is a fundamental issue in the conservation of materials used in Cultural Heritage sites, particularly those made of limestone. In this article, different facies of this stone were studied. The experiments, based on cycles of imbibition in a sodium sulfate solution and drying in a muffle furnace, aimed at measuring the progressive weight change from the initial weight of the sample. In this way, the resistance to salt crystallization could be assessed. It can be seen that the latter is strongly influenced by porosity and its size distribution, as well as by the mechanical properties of the material, which are consequently lower when porosity increases.

For each series of samples, the number of cycles at which weight loss relative to the initial mass occurred was measured. The onset of degradation due to salt crystallization was defined as the cycle at which 50% of the samples lost weight.

The reproducibility of the data is highly reliable, as, on average, 80% of the samples (as opposed to the 50% required by Flatt’s procedure) lost weight relative to the initial mass simultaneously and thus during the same salt crystallization cycle.

This determination was compared with that calculated through a chemomechanical model based on the conditions of salt crystallization and the properties of the material. The results demonstrate the notable reliability of the model in predicting the number of cycles of resistance to salt crystallization.

Although limited to the control of a small number of variables, the adopted procedure is confirmed to be capable of detecting the onset of degradation, in terms of weight loss, of the studied limestones and is proposed as a valuable tool to support researchers in the field of Cultural Heritage conservation. Further developments include the validation of this analytical procedure for different types of porous materials and, even more ambitiously, its incorporation into complex, multivariable models.

## Figures and Tables

**Table 1:** Materials properties for different facies: total porosity (ε), porosity in the first range (ε _r > 1 µm_), porosity in the second range (ε _1 < r < 0.05_ µm), porosity in the third range (ε _r < 0.05 µm_), average pore radius (APR), specific surface area (SSA) and bulk density (δ) and tensile strength (σ_T_).

Series	ε (%)	ε_r >1 µm_ (%)	ε_1 < r < 0.05 µm_ (%)	ε_r < 0.05 µm_ (%)	APR (µm)	SSA (m^2^/g)	δ (g/cm^3^)	σ_T_ (MPa)
C1	26.00 ± 2.00	12.08 ± 0.80	12.55 ± 0.45	1.37 ± 0.75	0.263 ± 0.62	1.14 ± 0.96	1.96 ± 2.00	4.0 ± 0.2
C2	30.26 ± 2.50	15.09 ± 0.73	13.95 ± 0.61	1.22 ± 1.16	0.275 ± 1.15	1.38 ± 1.08	1.95 ± 2.50	3.8 ± 0.3
C3	33.41 ± 2.30	16.65 ± 0.92	15.41 ± 0.75	1.35 ± 0.63	0.322 ± 0.53	1.39 ± 0.68	1.83 ± 2.30	3.2 ± 0.2
C4	36.81 ± 1.85	18.35 ± 0.83	16.97 ± 0.56	1.49 ± 0.46	0.321 ± 0.82	1.42 ± 0.94	1.76 ± 1.85	3.0 ± 0.4
C5	39.10 ± 2.20	19.96 ± 0.59	17.45 ± 0.43	1.69 ± 1.18	0.185 ± 0.79	1.86 ± 1.12	1.71 ± 2.20	2.8 ± 0.3
C6	43.38 ± 2.00	22.15 ± 0.74	19.36 ± 0.26	1.87 ± 1.00	0.213 ± 1.03	1.91 ± 0.94	1.58 ± 2.00	2.6 ± 0.6

## Data Availability

Data are contained within the article.
